# The importance of oriented physical activity in the first 48 months: differences in motor skills

**DOI:** 10.1186/s12887-023-04060-8

**Published:** 2023-05-11

**Authors:** Miguel Rebelo, João Serrano, Rui Paulo, Pedro Duarte-Mendes, Jorge Santos, Samuel Honório, João Petrica

**Affiliations:** 1 Department of Sports and Well-being, Polytechnic Institute of Castelo Branco, Rua Prof. Dr. Faria de Vasconcelos, Castelo Branco, 6000-266 Portugal; 2 Sport, Health & Exercise Research Unit (SHERU)- Polytechnic Institute of Castelo Branco, Castelo Branco, Portugal

**Keywords:** Motor Development, Motor skills, Physical activity, PDMS-2

## Abstract

**Background:**

The importance of physical activity in the first months of age is well known, however, with the evolution of the urban environment, the excessive workload of parents and the excessive time in growing up in kindergartens has limited this same free practice and little has been studied about this issue. In Portugal, there are institutions that provide oriented physical activity for their children, however, this is optional, which may create disadvantages in children’s motor skills in these ages.

**Objective:**

The objective of the study isto verify if there are differences in the development of motor skills (global and fine) comparing children between 12 and 48 months who practice oriented physical activity (OPA) and children who do not.

**Methods:**

Participated in this study, 400 children of both genders (28.14 ± 7.23 months). Two groups were created (the group that had oriented physical activity (30 min long and 2 times a week) and the group that didn’t have oriented physical activity). For a better understanding they were divided into 3 age groups (12–23, 24–35 and 36–48 months). Motor skills were assessed using the PDMS-2 scales, for 6 months, following the instrument’s application standards.

**Results:**

In a first analysis, we found that the majority of children only start to practice oriented physical activity in institutions from 36 months of age, however, it is in the first months (from 12 to 35) that the greatest differences between the two groups can occur. The OPA group presented better results according to the mean values, in all motor skills. Differences between groups were most noticeable in Postural, locomotion and fine manipulation Skills (showing effect size: moderate and low).

**Conclusions:**

We can conclude that a practice of oriented physical activity in the first 48 months is fundamental to the development of motor skills. It is in the first months (up to 36) that there are greater differences, but it is also where there are less children carrying out guided physical activity. This is an important factor, and is determinant to make institutions aware of this importance of this variable in child development.

**Supplementary Information:**

The online version contains supplementary material available at 10.1186/s12887-023-04060-8.

## Background

Motor development presupposes a set of change processes that last throughout life, mainly occurring mostly during the first years of life, with each child having different developmental rhythms [1]. According to several authors, the practice of physical activity is one of the most critical factors, because, according to Neto [2] and Barnett et al. [3] physical activity is a matter of education and as such, the school is one of the best places to develop promotion strategies, noting that it is essential to provide it with guidance and according to the individual characteristics of each child.

According to Fernandes [4] and Messerli-Bürgy et al. [5] it is vital for children to practice physical activity, not only to improve their physical shape but also for reasons related to health, socialization, and school and also because it is an activity linked to their well-being. The same authors refer in their studies, arguing that physical activity is a complex set of behaviors that accompany any body movement, produced by skeletal muscles, and which mainly result in an increase in energy expenditure above resting levels.

On the other hand, Newell [6] defines physical activity more narrowly, defining it as an intentional voluntary movement performed to achieve an identifiable goal. In this same line of thought Maia and Lopes [7] say that physical activity is understood as a complex behavior involving some variables, namely, duration, frequency, intensity and type. As Riddoch and Boreham [8] share the same opinion stating that physical activity represents a very complex behavior, which can vary within the range of dimensions such as: type of activity, session duration, intensity and session program.

However, little is known about which types of physical activity are more effective and in which contexts for a better development of motor skills, being fundamental further investigations on this subject [9], and more longitudinal research is needed to investigate and confirm the benefits potentials of structured leisure-time PA for the development of motor skills [10–12].

According to Santos, Dantas and Oliveira [13], Motor Development in the first years of life is characterized by the acquisition of a vast repertoire of motor skills (global and fine), which enables the child to have complete mastery of his body in different postures, moving around the environment in different ways (walking, running, jumping, etc.) and manipulating different objects and instruments (receive a ball, throw a stone, kick, write, etc.). The same author, these basic skills are required for carrying out daily routines at home and at school, as well as serving ludic purposes, always present in childhood.

Regarding the instrument used to collect data in this study, global motor skills are observed in the participation of large muscle groups that produce strength in the upper-body, arms and legs [14]. These types of movements are easier for a child to control and is generally develop faster than fine motor skills. Some of the movements that are considered global motor skills are: running, jumping, kicking, rolling and dancing [10]. As for Fine motor skills, they are observed by carrying out movements of the small muscles of the body [13], referring to the ability to coordinate specific movements of some segments of the body, to achieve a very precise result according to the task (for example: writing, painting, perforating, among others).

For the World Health Organization [WHO] (2007), the benefits of an active childhood can be transferred to adulthood; thus, an active child is more predisposed to become an active adult.

Mainly in the last decade, there has been a significant decrease in the time spent by children in relation to free exploration, contact with nature, spontaneous play, contact with friends and physical activity initially supervised and oriented. In this line of thought, Valentini et al. [13] argues that, lately, children have very busy schedules, as well as the constraints of lack of space and mobility existing in the life of urban cities, towns and villages, the lack of risk and adventure and a healthy diet are progressively creating an obese, sedentary and motor-illiterate generation, with very serious short-term consequences for public health.

The importance of physical activity for human beings is a permanent theme in contemporary society. The child develops thanks to his motor skills, which intimately modify and organize his nervous system. We also understand that in an initial phase, the primary means of promoting the development and practice of movement, of playing, is through the exploration of the environment/space. Furthermore, in this sense, everything depends on parental involvement. However, this involvement increasingly defends the spirit of protection, in which it is safer for children to be quiet/trapped, than allowing them to have the possibility to explore the environment and space, and this can create several risks, which clearly parental involvement is not prepared for [13].

Faced with this problem, and since it is in daycare centers and kindergartens where children spend most of their time, it is essential to create and offer children opportunities to practice physical activity, whether through explorations of spaces or through supervised and/or oriented physical activity. Agreeing with this idea, Seabra [15] attributes its main relevance to schools, stating that they are responsible for implementing programs to promote physical activity.

It is thus relevant to know if children who have physical activity and/or physical-motor expression classes in the first months of life have better results in motor skills and if these results may or may not compromise the development processes, creating inequalities within the respective groups. Thus, this study aimed to verify if there were differences in the development of motor skills (global and fine) comparing children who have or do not have oriented physical activity in their institutions.

## Subjects and method

### Subjects

This cross-sectional study was carried out with 400 children of both genders aged (28.14 ± 7.23 months) between 12 and 48 months (F = 203, 28.35 ± 7.99 months; M = 197, 29.14 ± 7.73 months). All these children live in the urban environment in a town of Portugal. For better analysis and understanding of the results, the participants were divided into three groups by age group: from 12 to 23 months (N = 104, age = 17.79 ± 3.33 months), from 24 to 35 months (N = 152, age = 28.27 ± 3.65 months) and from 36 to 48 months (N = 144, age = 17.35 ± 4.16 months).

These children were grouped as follows: children with oriented physical activity (N = 190, age = 33.96 ± 6.99 months); children without oriented physical activity (N = 215, age = 25.83 ± 8.54 months). The oriented physical activity classes/activities had a duration of 30 min and twice a week, and the respective motor skills were analyzed depending on age level.

Initially, a contact was established with institutions, daycare centers and/or kindergarten, with which the Polytechnic Institute of Castelo Branco has collaboration protocols for possibility of applying the instrument to children; with 730 children being contacted, that only 400 agreed to participate in the study (55%), taking 2 months to obtain authorizations from parents and/or guardians and later data collection took another 6 months to be carried out for the entire number of participants.

The following exclusion criteria were considered: Children diagnosed with learning difficulties and/or developmental impairments; Children with some type of diagnosed disability; Children under 12 months and over 48 months.

.

### Instruments

The instrument used to collect information on the motor skills of the children under study was the Peabody Developmental Motor Scales - Second Edition (PDMS-2) [14]. The PDMS-2 scales are one of the most used instruments in the scope of motor assessment. The scales were reviewed by Saraiva and Rodrigues [16] and Rebelo et al. [17] for the Portuguese population (χ2 = 55.614; df = 4; p = .06; χ2/df = 13.904; SRMR = 0.065; CFI = 0.99, TLI = 0.99; α = 0.85 and ICC = 0.98) and allow assessing the performance of fine and global motor skills in children from birth to 71 months.

The results of the PDMS-2 are indicated in three domains of motor behavior, the fine motor quotient (FMQ), the global motor quotient (GMQ) and the total motor quotient (TMQ) that results from the previous two. The scale presents the child’s global motor profile, as well as the result of the motor sub-tests that gather the entire scale [15]. The scale presents the child’s global motor profile, as well as the result of the motor subtests that make the scale completed [14].

The items are summed in each of the tests and their value is located in the reference table for age, resulting in a standardized value and a percentile value that can be compared between ages. Subsequently, the sum of the standardized values of the grouped tests makes it possible to obtain the TMQ, GMQ and FMQ according to the consultation of an appropriate table. Finally, the standardized values can be converted into a qualitative classification with categories (from Very Weak to Very Good), shown in supplementary table (S1) [14].

The scales are standardized for the child population and have a mean value of 10 points (± 3) for each test and a mean value of 100 (± 15) for the motor quotients [14].

The results of each test can be expressed through 5 types of final scores: raw score; equivalent age score; standard score or Z-score; percentiles and motor quotients. These scores are the most important information associated with the child’s performance. Its analysis provides additional information from the test, which together with other knowledge acquired through other sources results in a good diagnosis of the child’s problem [14].

To obtain information about the participants, a child characterization form was created, in which information was collected on whether they practiced or not physical activity in their institutions.

### Procedures

After approval by the institution for data collection, an informed consent form was sent to parents and the child’s characterization form was requested to be completed, which allowed us to select the children considering the exclusion requirements of the study. All ethical principles, norms and international standards relating to the Declaration of Helsinki and the Convention on Human Rights and Biomedicine were followed, respected and preserved [16]. This project was approved by the institution’s Ethics Committee, where the authors conduct their research.

According to Folio and Fewell [16], examiners who use the PDMS-2 as an assessment instrument must: understand the general procedures for administering the test, its rating and interpretation, for which pilot assessments/studies were carried out to adapt to the instruments. A single researcher, a specialist in the area of motor development, collected data.

The administration of PDMS-2 was individual and applied for approximately 45 to 60 min in a room or a large space with stairs. The assessment place was previously prepared in order to provide an environment with the least amount of stimuli and distractions possible considerer to disturb children. The test application time respected the daycare routines, meal, bath and sleeping times. When interrupted, assessments were completed within five days, as established by scale authors [16].

In order to correctly administer the instrument, the following rules were followed: Instructions were repeated to the child three times in order to provide the opportunity to reach the maximum score in each item; the child started the test at a point on the scale established by their age (these points were empirically determined to allow the examiner to start the test on an item that 75% of the children in the normative sample of that age passed) proceeding in the sequence until the test fails of three consecutive items. The score for each item is 0 to 2 (0 does not perform, 1 performs with difficulty, and 2 performs well) [15]. After the evaluation, the sum of each item is calculated until the final result is established in global, fine and total motor skills (which is the sum of global and fine skills). Subsequently, the value of the sum of the items, in each of the subscales is located in a reference table for age, where a standardized value is obtained (from 1 to 20), which can be converted into a qualitative classification with seven categories (from Very Good” to “Very Weak) [16].

### Statistical analysis

We used the IBM - SPSS - Statistical Package for the Social Sciences SPSS (v.23.0). In the first analysis, the Kolmogorov-Smirnov test verified the sample normality. As we obtained a non-normal distribution (*p*. <0.05) for all variables under study, we used the Mann-Whitney U test for independent samples, which allowed us to assess the differences between groups. The method of inferences based on the magnitude of the effects was also performed, using the following scale (d Cohen): 0-0.2, trivial; 0.21–0.6, low; 0.61–1.2, moderate; 1.21-2.0, high; 2.0, very high [19].

## Results

As for the general characterization of the participants (Table 1), it was in postural skills (10.99 ± 1.39; 11.75 ± 1.57; 12.53 ± 2.14) that children show better results in all age groups (12–23; 24–35; 36–48 months), however, children have more difficulties in locomotion skills (7.61 ± 1.25; 8.69 ± 1.69; 9.14 ± 1.10).

It is only in the age group from 12 to 23 months that global motor skills (97.12 ± 8.14 vs. 95.88 ± 8.81) show better results, since in the remaining age groups it is fine motor skills (24–35 months, GM = 98.33 ± 8.70 vs. FM = 98.01 ± 11.02; 36–48 months, GM = 110.39 ± 11.71 vs. FM = 101.81 ± 7.98) which presents better results in these children.

**Table 1:** Overall average(M) and standard deviation (SD) of the sample obtained in the subtests by age group

Subtests		12–23 Months (n = 104)	24–35 Months (n = 152)	36–48 Months (n = 144)
**Postural Skills**	M ± SD	10.99 (1.39)	11.75 (1.57)	12.53 (2.14)
**Locomotion Skills**	M ± SD	7.61 (1.25)	8.69 (1.69)	9.14 (1.10)
**Object Manipulation Skills**	M ± SD	9.75 (1.5)	9.05 (1.8)	9.12 (1.67)
**Fine manipulation skills**	M ± SD	10.11 (1.8)	10.71 (1.7)	11.31 (2.7)
**Visuomotor integration skills**	M ± SD	8.97 (1.83)	9.91 (1.23)	10.91 (2.8)
**Global Motricity**	M ± SD	97.45 (8.14)	98.9 (11.1)	105.02 (8.8)
**Fine Motricity**	M ± SD	96.78 (8.81)	99.89 (11.1)	109.78 (8.8)

Table 2 presents the results of the comparative analysis within each age range between children who had oriented physical activity at the institution and those who did not have any oriented physical activity practice at the institution. In the first analysis, we found that a large majority of children only begin to practice-oriented physical activity in institutions from the age of 36 months. However, in the first years (from 12 to 35) there are more statistically significant differences between the two groups, presented in all variables. The group that practices oriented physical activity presents, on average, better results in all motor skills.

In the 12–23-months age range, we can observe that there were significant differences in postural skills (*p* < .001; η^2^ = 0.133; effect size: moderate), locomotion skills (*p* = .043; η^2^ = 0.036; effect size: low) and fine manipulation skills (*p* = .007; η^2^ = 0.064; effect size: low), with the group of children who practice-oriented physical activity obtaining better results. It is important to emphasize regarding the qualitative analysis that although there were no statistically significant differences in all other variables, this same group presented, on average, the best results.

The age ranges from 24 to 35 months, was the one that presented more skills with statistically significant differences, locomotion (*p* = .036; η^2^ = 0.027; effect size: low), fine manipulation (*p* = .021; η^2^ = 0.033; effect size: low), visuomotor integration (*p* < .001; η^2^ = 0.075; effect size: low) and fine motricity (*p* < .001; η^2^ = 0.102; effect size: moderate), also with the group with physical activity oriented to obtain better results in motor skills.

At the group age of 36 to 48 months, this is where there are the least statistically significant differences, only in fine motricity (*p* = .039; η^2^ = 0.029; effect size: low), with the groups of physical activity practice-oriented to obtain better results, as happens, in qualitative analysis, in all as remaining variables.

**Table 2:** Differences regarding the sibling presence variable in the PDMS-2 for each age range

Age Range	PDMS-2	Oriented physical activity practice	N	M ± SD	*p*	*η* ^*2*^	Effect Size	
12–23 months	Postural skills	Yes	18	**12.39 ± 1.54**	< 0.001*	**0.133**	**0.782**	
		No	86	10.71 ± 1.80				
	Locomotion skills	Yes	18	**7.71 ± 1.24**	0.043*	**0.036**	**0.385**	
		No	86	7.06 ± 1.21				
	Object manipulation skills	Yes	18	10.35 ± 2.69	0.239	0.013	0.226	
		No	86	9.50 ± 0.51				
	Fine manipulation skills	Yes	18	**10.50 ± 1.54**	0.007*	**0.064**	**0.522**	
		No	86	9.27 ± 2.15				
	Visuomotor integration skills	Yes	18	9.64 ± 1.75	0.378	0.007	0.167	
		No	86	8.83 ± 2.68				
	Global motricity	Yes	18	97.89 ± 2.70	0.520	0.004	0.123	
		No	86	96.97 ± 8.84				
	Fine motricity	Yes	18	98.00 ± 8.85	0.501	0.004	0.128	
		No	86	95.45 ± 8.79				
24–35 months	Postural skills	Yes	64	11.91 ± 1.49	0.213	0.008	0.184	
		No	88	11.64 ± 1.63				
	Locomotion skills	Yes	64	**9.02 ± 1.64**	0.036*	**0.027**	**0.332**	
		No	88	8.45 ± 1.69				
	Object manipulation skills	Yes	64	9.22 ± 1.40	0.113	0.016	0.254	
		No	88	8.67 ± 2.13				
	Fine manipulation skills	Yes	64	**10.55 ± 1.78**	0.021*	**0.033**	**0.368**	
		No	88	9.85 ± 1.59				
	Visuomotor integration skills	Yes	64	**10.00 ± 8.91**	< 0.001*	**0.075**	**0.570**	
		No	88	8.91 ± 1.93				
	Global motricity	Yes	64	99.98 ± 7.71	0.526	0.003	0.102	
		No	88	96.69 ± 12.75				
	Fine motricity	Yes	64	**101.64 ± 9.04**	< 0.001 *	**0.102**	**0.673**	
		No	88	95.96 ± 7.65				
36–48 months	Postural skills	Yes	107	12.42 ± 2.15	0.240	0.009	0.191	
		No	37	12.89 ± 2.08				
	Locomotion skills	Yes	107	9.16 ± 1.07	0.205	0.010	0.204	
		No	37	9.08 ± 1.21				
	Object manipulation skills	Yes	107	8.83 ± 1.54	0.152	0.013	0.233	
		No	37	9.22 ± 1.62				
	Fine manipulation skills	Yes	107	12.45 ± 2.69	0.067	0.023	0.305	
		No	37	11.78 ± 1.93				
	Visuomotor integration skills	Yes	107	11.28 ± 2.24	0.243	0.009	0.192	
		No	37	10.84 ± 2.22				
	Global motricity	Yes	107	108.60 ± 7.93	0.647	0.001	0.069	
		No	37	102.41 ± 9.18				
	Fine motricity	Yes	107	**111.25 ± 12.32**	**0.039**	**0.029**	**0.347**	
		No	37	107.86 ± 9.39				

* *p* ≤ .05 using the Mann–Whitney U test; significant *p*-values and their associated effects are in bold. N—Number of Subjects; M—Mean; SD—Standard Deviation

## Discussion

The objective of the present study was to verify whether children who practiced guided physical activity in institutions had better results in global and fine motor skills in the first 48 months of life, considering different age groups.

In global terms, it was verified that children of these ages show more capabilities in postural skills, as opposed to locomotion skills, which is where children are more lagging behind in development, as happened in the studies by Rebelo [20], Logan et al. [21] and Gaul and Issartel [22], as the same authors also refer about children of these ages already showing better results in fine motor skills compared to global motor skills. This has already been mentioned by several authors [5, 12, 21, 22], that children’s preferences in the last decade for video games has brought detriment of free play in the street, which in itself in the first years of life causes a natural delay in global motor development, mainly locomotor skills, such as walking, running and jumping.

As for the variable studied, it has been shown to influence the development of motor skills in the group of children with physical activity oriented at the institution, where, on average, children in this group obtained better results in all motor skills and in different age groups as happened in the study of Dapp, Gashaj and Roebers [9], in which, in his longitudinal study, he concluded that guided physical activity is a promising way to promote the development of children’s motor skills in the long term.

Despite the few studies that investigate the importance and influence of physical activity in these age groups [9], our results are in line with most investigations, stating that physical activity is essential for children, not only to improve their physical fitness but also for issues related to health, socialization, and their well-being as O’ Brien, Belton and Issartel [23] and Neto [2] defends the regular practice of regular physical activity in younger children, which contributes to immense benefits for the development, whether of physical motor skills, or the creation of new friendships and appreciation of self-esteem, with special emphasis between 12 and 35 months, since it is in this age group that there are more differences, which by consequence is where there are fewer children with this type of practice.

These results also aim to alert institutions so that all children have the right to guided physical activity, knowing today that 70% of children’s time is spent in the respective institutions, which is why, according to Seabra [15], institutions (nurseries and kindergartens) are responsible for implementing programs to promote physical activity.

Our results also corroborate those of Stodden et al. [24] who mentions that the practice of supervised and/or oriented physical activity in daycare centers, preschool and elementary school, plays a relevant role, allowing improvements in the global motor development of children.

An interesting fact in the results obtained is that there is a big difference, mainly in fine motor skills, in children who have oriented physical activity, which highlights the fact that these classes/activities do not only give importance or value to global motor skills, also promoting fine motor skills and contributing to the harmonious multilateral and interdisciplinary development of all motor skills.

Thus, it becomes increasingly important that daycare centers and kindergartens give due importance and that the practice of physical activity becomes a curricular activity for all children, even in this age group, because according to Neto [2] lately, children have very busy schedules, as well as the constraints of lack of space and mobility existing in the life of cities, towns and villages, the lack of risk and adventure and an unhealthy diet, being progressively obese, sedentary and motor-illiterate, with very serious short-term consequences for public health. As for Rocha, Campos and Rocha [25], it is in the periods when the child attends kindergarten that the sensitive phases to the learning of specific skills and abilities occur, so the development of this same practice becomes essential and fundamental.

Some of the limitations of the present study were: (a) the veracity and typology of the type of exercises performed in the respective classes; (b) the influence of other variables that may influence the results; (c) the fact that this is a cross-sectional study, which does not allow us to draw causal conclusions.

Thus, in future studies, it is suggested to replicate the same with samples from other regions, as well as the relationship with other variables that may influence motor development.

## Conclusions

Globally, we can conclude that the variable oriented physical activity in the first 48 months has an influence on the development of motor skills, with children with this orientation showing better results in several motor domains. It should also be noted that it is in the first months (up to 36) that there are greater differences, but it is also where there are fewer children carrying out oriented physical activity. This is an important factor in making institutions aware of the importance of carrying out these activities in the first months, emphasizing that these are a privileged moment to stimulate children’s fundamental motor skills.

## Electronic supplementary material

Below is the link to the electronic supplementary material.

Supplementary Material 1

## Data Availability

Authors can confirm that all relevant data are included in the article, and, the authors declare that [the/all other] data supporting the findings of this study are available within the article.
