# Supplementary material for: The importance of oriented physical activity in the first 48 months: differences in motor skills
Source: BMC Pediatr. 2023 May 11;23:232. doi: 10.1186/s12887-023-04060-8 (PMC10173640; doi:10.1186/s12887-023-04060-8)
Supplement: Supplementary file 1 — Supplementary Material 1 [file 12887_2023_4060_MOESM1_ESM.doc]

**Supplementary table1. The Peabody Developmental Motor Scales—Second Edition (PDMS-2) subtest standard score values with the associated classification/description.**

| **Standard Scores** | **Classification** |
| --- | --- |
| 17-20 | Very Good |
| 15-16 | Good |
| 13-14 | Above Average |
| **8-12** | **Average** |
| 6-7 | Below Average |
| 4-5 | Weak |
| 1-3 | Very Weak |
